# DHCR24, a Key Enzyme of Cholesterol Synthesis, Serves as a Marker Gene of the Mouse Adrenal Gland Inner Cortex

**DOI:** 10.3390/ijms24020933

**Published:** 2023-01-04

**Authors:** Huifei Sophia Zheng, Yuan Kang, Qiongxia Lyu, Kristina Junghans, Courtney Cleary, Olivia Reid, Greer Cauthen, Karly Laprocina, Chen-Che Jeff Huang

**Affiliations:** Department of Anatomy, Physiology & Pharmacology, College of Veterinary Medicine, Auburn University, Auburn, AL 36849, USA

**Keywords:** adrenal gland inner cortex, X-zone, DHCR24, seladin-1

## Abstract

Steroid hormones are synthesized through enzymatic reactions using cholesterol as the substrate. In steroidogenic cells, the required cholesterol for steroidogenesis can be obtained from blood circulation or synthesized de novo from acetate. One of the key enzymes that control cholesterol synthesis is 24-dehydrocholesterol reductase (encoded by *DHCR24*). In humans and rats, DHCR24 is highly expressed in the adrenal gland, especially in the zona fasciculata. We recently reported that DHCR24 was expressed in the mouse adrenal gland’s inner cortex and also found that thyroid hormone treatment significantly upregulated the expression of *Dhcr24* in the mouse adrenal gland. In the present study, we showed the cellular expression of DHCR24 in mouse adrenal glands in early postnatal stages. We found that the expression pattern of DHCR24 was similar to the X-zone marker gene 20αHSD in most developmental stages. This finding indicates that most steroidogenic adrenocortical cells in the mouse adrenal gland do not synthesize cholesterol locally. Unlike the 20αHSD-positive X-zone regresses during pregnancy, some DHCR24-positive cells remain present in parous females. Conditional knockout mice showed that the removal of *Dhcr24* in steroidogenic cells did not affect the overall development of the adrenal gland or the secretion of corticosterone under acute stress. Whether DHCR24 plays a role in conditions where a continuous high amount of corticosterone production is needed requires further investigation.

## 1. Introduction

**Figure 1:**
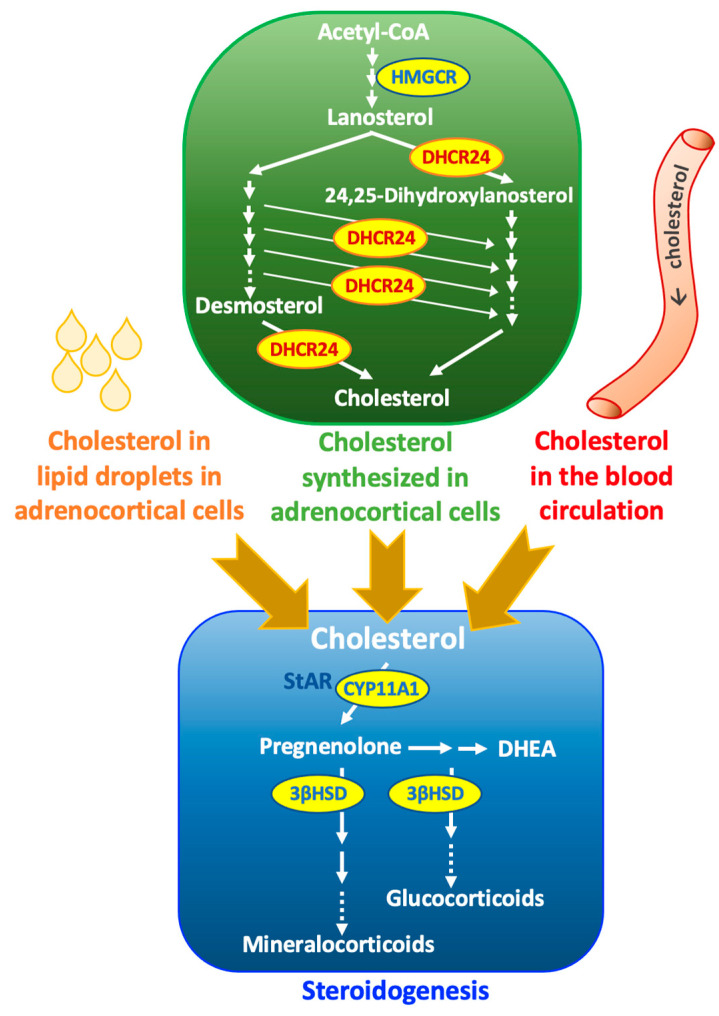
Cholesterol synthesis and steroidogenesis in the adrenal gland.

The mitochondrial cytochrome P450 cholesterol side-chain cleavage enzyme (P450scc, encoded by *CYP11A1*) controls the first step of the steroidogenesis pathway, which converts cholesterol into pregnanolone (Figure 1) [1]. Steroidogenic cells can obtain the required cholesterol for steroidogenesis by taking up circulating cholesterol via receptors on the cell membrane, using the cholesterol stored in lipid droplets in the cytoplasm, or synthesizing cholesterol de novo from acetate (Figure 1) [2]. Similar to many other steroid-producing cells, adrenocortical cells can synthesize cholesterol locally [3]. In human adrenal glands, de novo synthesized cholesterol contributes to 20% of cortisol production [4]. One of the key enzymes that control cholesterol synthesis is DHCR24, which is highly expressed in the adrenal gland in both humans [5] and rats [6], especially in the zona fasciculata. *DHCR24* is also named Selective Alzheimer disease indicator 1 (seladin-1) because it was first identified using neuronal cells from Alzheimer’s disease (AD) patients [7]. In humans, the adrenal gland is the tissue with the highest expression of DHCR24 [7]. The expression of DHCR24 has been reported to be altered in human adrenal adenomas and carcinomas [8,9]. Patients carrying mutations in DHCR24 show the accumulation of cholesterol precursor, desmosterol, and cause desmosterolosis, which is a disorder characterized by multiple congenital anomalies, neurological complications, and developmental delays [10]. We recently reported that DHCR24 was expressed in the mouse adrenal gland inner cortex partially colocalized with the inner cortex marker gene 20αHSD [11]. In mouse adrenal glands, the 20αHSD-positive zone is also the X-zone [12], a structure that undergoes regression during postnatal development [13]. A recent study used single-cell approaches and chronic stress challenges to demonstrate the existence and the recruitment of a subpopulation localized in the adrenal gland inner cortex [14]. This finding suggests that the inner part of the adrenal cortex might be critical for optimal stress response. To characterize the significance of the zonal-restricted marker gene, DHCR24, in the mouse adrenal gland inner cortex, we used X-gal staining, immunostaining, and quantitative polymerase chain reaction (qPCR) for the time-course expression of DHCR24. A conditional knockout (cKO) mouse model was used to define the possible role of DHCR24 in adrenal gland development and function at the baseline level (without treatments/challenges other than CO_2_ euthanization).

## 2. Results

### 2.1. Cellular Expression of DHCR24 in Mouse Adrenal Glands

**Figure 2:**
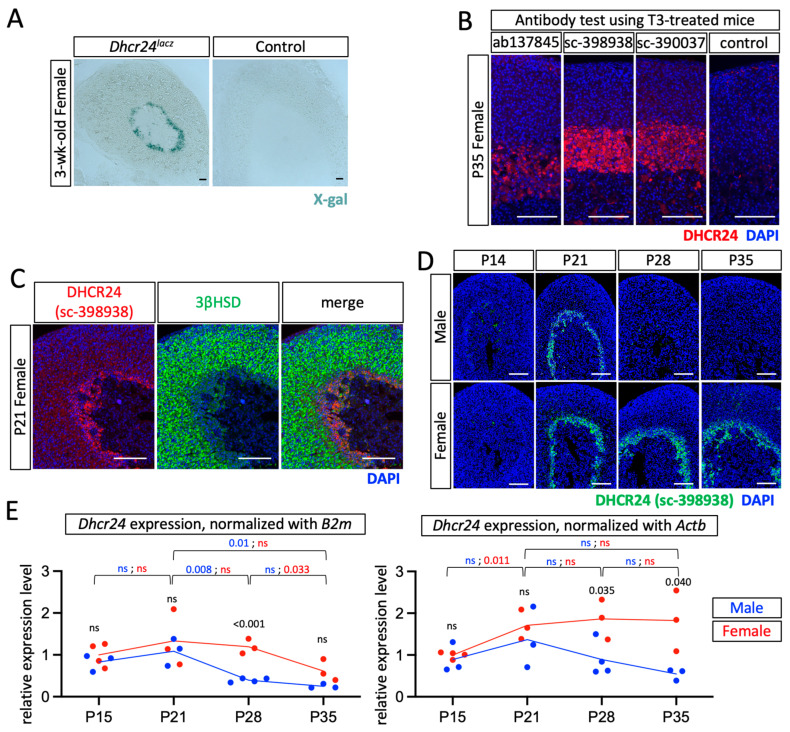
DHCR24 expression. (**A**) X-gal staining of tissues from 3-week-old female mice that were either heterozygous for *Dhcr24^lacz^* or the wild-type from the *Dhcr24^lacz^* strain. (**B**) Immunostaining of DHCR24 using three different commercially available antibodies. Mice were treated with T3 water for 10 days to increase the expression level of DHCR24. The negative control sample was stained using the same method except for incubating with the primary antibody. (**C**) Double immunostaining using DHCR24 antibody #sc-398938 and 3βHSD on euthyroid wild-type B6 mice. (**D**) Immunostaining using DHCR24 antibody #sc-398938 on euthyroid wild-type B6 mice. (**E**) Quantitative polymerase chain reaction was performed to detect the relative expression levels of *Dhcr24*, normalized with either *B2m* or *Actb* to P15 males (set as 1), in whole adrenal glands. *p*-values are shown for the comparison of the same sex between adjacent time points (font color red: female, font color blue: male), and the comparison of male vs. female within each time point (font color black). The trends are shown with the mean. Each data point contains pooled samples from at least three mice. The cell nuclei were counterstained with DAPI (blue). Scale bars, 130 µm. ns, no significant difference.

**Figure 3:**
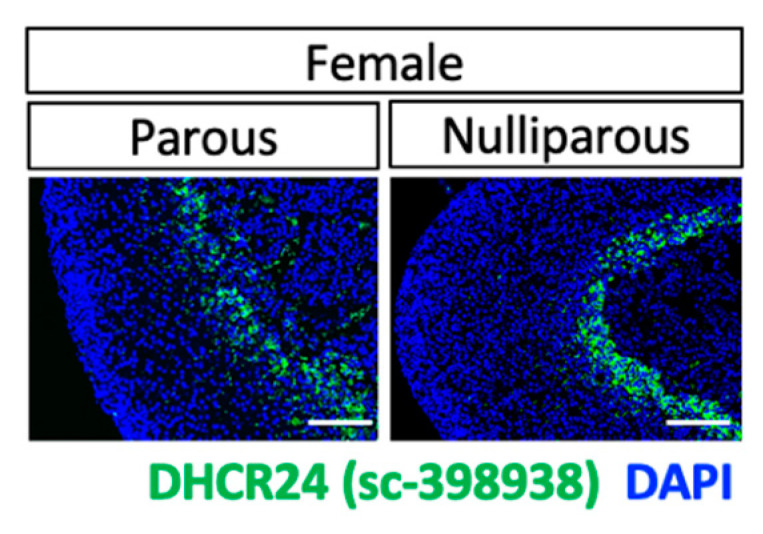
Immunostaining using DHCR24 antibody #sc-398938 of tissue from parous and nulliparous euthyroid wild-type B6 mice. The cell nuclei were counterstained with DAPI (blue). Scale bars, 130 µm.

A previous finding of the expression of DHCR24 in the mouse adrenal gland inner cortex [11] was confirmed by X-gal staining (Figure 2A). Three commercially available antibodies against DHCR24 were able to detect DHCR24 on formalin-fixed, paraffin-embedded adrenal gland sections (Figure 2B) from mice that were treated with T3, which is known to significantly induce DHCR24 expression [11,15,16] through thyroid hormone receptor (TR) binding sites on the promotor region [17]. Under the euthyroid condition, the DHCR24-positive cells were mainly in the 3βHSD low-expressing inner cortex (Figure 2C), which is also the X-zone in mouse adrenal glands [12]. To obtain the spatial and temporal expression profile of DHCR24 in mouse adrenal glands, both male and female mice were analyzed by immunostaining (Figure 2D) and qPCR (Figure 2E). Both results showed that DHCR24 was expressed in postnatal adrenal glands with a sexually dimorphic pattern. The sexually dimorphic cellular expression and the expression timeline were similar to those of the X-zone marker gene 20αHSD [11,18]. At P14, immunostaining results showed that a few DHCR24-positive cells were found at the cortical–medullary boundary in both sexes. A clear DHCR24-positive zone existed in the inner-cortical region at P21. At P28 and P35, the DHCR24-positive zone remained in adrenal glands in female mice. However, in male mice, only a few DHCR24-positive cells were found at P28; immunostaining did not detect any DHCR24-positive cells in males at P35 (Figure 2D). Despite the strong increase seen by immunostaining from P14 to P21 in both sexes, it is interesting that the qPCR result only showed an increase of the *Dhcr24* expression in female mice when the expression is normalized with *Actb*. It is possible that a housekeeping gene is differentially expressed across different postnatal stages [19], thus masking the differential expression of *Dhcr24*. Unlike the 20αHSD-positive zone that regresses soon during the first pregnancy [13], immunostaining showed that the DHCR24-positive zone remained present in parous females (Figure 3), suggesting that pregnancy does not lead to loss of the DHCR24-positive zone in the adrenal glands of female mice.

### 2.2. Deletion of Dhcr24 in Steroidogenic Cells Did Not Affect the Zonal Structure of the Adrenal Gland

**Figure 4:**
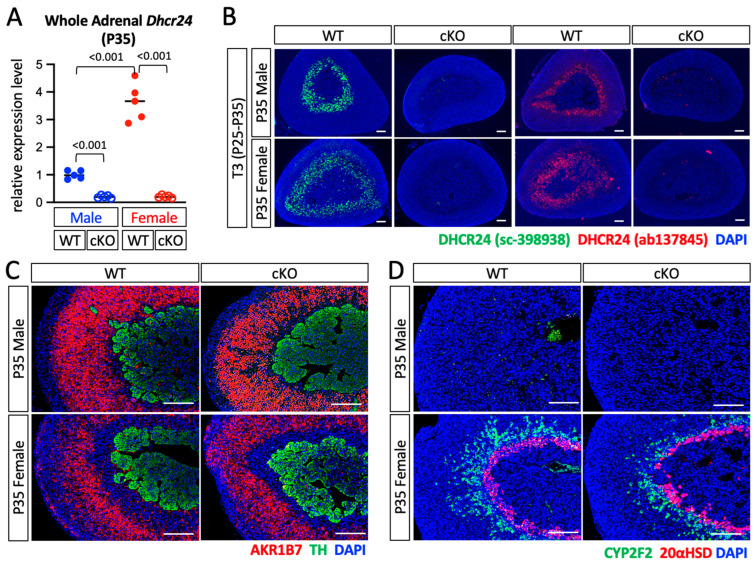
Phenotypic analyses of *Dhcr24* cKO mice. (**A**) Quantitative polymerase chain reaction was performed to detect the relative expression levels of *Dhcr24* in whole adrenal glands from cKO and WT littermates at P35. The relative expression levels of *Dhcr24* in cKO mice were normalized to those of the male WT mice. Each dot (WT) and circle (cKO) represents data from one mouse. Data are shown with the mean. (**B**) Immunostaining using DHCR24 antibodies #sc-398938 and #ab137845. The mice were treated with T3 drinking water for 10 days to increase the expression level of DHCR24. The cell nuclei were counterstained with DAPI (blue). (**C**,**D**) Immunostaining showed areas of zona fasciculata (AKR1B7-positive), medulla (tyrosine hydroxylase (TH)-positive), and inner cortex (CYP2F2- or 20αHSD-positive) in euthyroid P35 mice. The cell nuclei were counterstained with DAPI (blue). WT: wild-type mice, cKO: *Dhcr24* conditional knockout mice. Scale bars, 130 µm.

**Figure 5:**
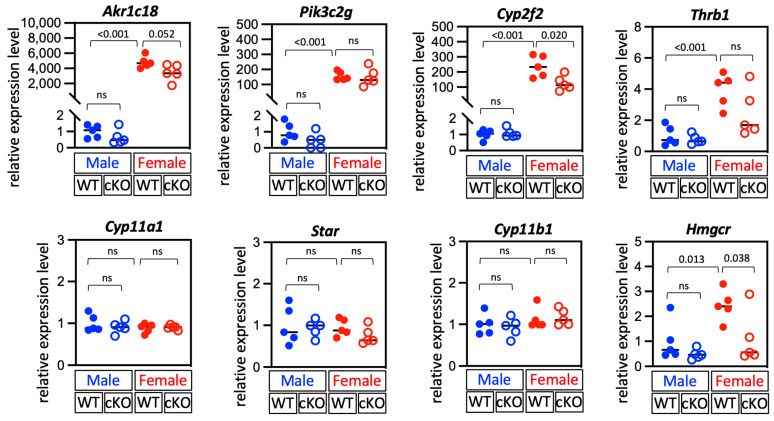
Quantitative polymerase chain reaction showed the relative expressions of key marker genes in the adrenal cortex. The relative expression levels of each gene in cKO mice were normalized to those of the male WT mice. Each dot (WT) and circle (cKO) represents data from one mouse. Data are shown with the mean. WT: wild-type mice, cKO: *Dhcr24* conditional knockout mice. ns, no significant difference.

To understand the role of DHCR24 in the adrenal gland, we used *Sf1-Cre* to remove *Dhcr24* in steroidogenic cells. The expression of *Dhcr24* in the whole adrenal gland shown by qPCR was dramatically reduced in cKO mice (*Dhcr24^flox/flox^; Sf1Cre*) in both sexes (Figure 4A). The expression level of *Dhcr24* in P35 cKO males was 24.6% of that in wild-type males, whereas in female cKO mice at the same age, the expression was only 10.2% of that in wild-type females. An RNA-seq result in a previous study showed that the expression levels of *Dhcr24* in the whole adrenal gland in P35 mice were 33.62 FPKM (fragments per kilobase of exon per million mapped fragments) in males and 111.96 FPKM in females [11]. The reduction rates of the *Dhcr24* expression in cKO mice shown by the qPCR suggested that the expression levels of *Dhcr24* in the adrenal glands in cKO mice could be around 10 FPKM in both sexes. This finding indicates that *Dhcr24* is mainly expressed in SF1-positive cortical cells and that *Sf1-Cre* could remove most *Dhcr24* in the adrenal gland. Indeed, immunostaining showed that no DHCR24-positive cells were detected in adrenal glands in cKO mice of both sexes in the euthyroid condition. Even after thyroid hormone treatment, the adrenal glands in cKO mice remained DHCR24-negative for immunostaining signals (Figure 4B), confirming the deletion of DHCR24 in cKO mice adrenal glands. To determine if the structure of the concentric cortical layers was affected in cKO adrenal glands, we performed immunostaining to label different cortical layers, including the X-zone and the CYP2F2-positive zone, which is a newly identified inner subzone in mouse adrenal zona fasciculata [11]. The lack of DHCR24 did not lead to any significant change in adrenal cortex zonation at P35 (Figure 4C,D). The qPCR results showed that expressions of three major marker genes of the inner cortex, *Akr1c18* (encodes 20αHSD), *Pik3c2g*, and *Thrb1* were not significantly altered in cKO adrenal glands (Figure 5). Interestingly, the *Cyp2f2* expression was slightly reduced in female cKO adrenal glands (WT vs. cKO: 1 ± 0.30 vs. 0.53 ± 0.20, *p* = 0.020). Expressions of *Cyp11a1* and *Star*, the two key factors that control the rate-limiting step of steroidogenesis, were not affected by the deletion of *Dhcr24*. The mRNA level of the steroidogenic enzyme 11β-hydroxylase (encoded by *Cyp11b1*) was also unchanged. The enzyme that controls the rate-limiting step of cholesterol synthesis (HMG-CoA reductase, encoded by *Hmgcr*), was slightly reduced in cKO females (WT vs. cKO: 1 ± 0.26 vs. 0.45 ± 0.43, *p* = 0.038).

### 2.3. Deletion of Dhcr24 in Steroidogenic cells Did Not Affect the Lipid-Droplet Accumulation in Adrenocortical Cells or the Blood Corticosterone and ACTH Levels

**Figure 6:**
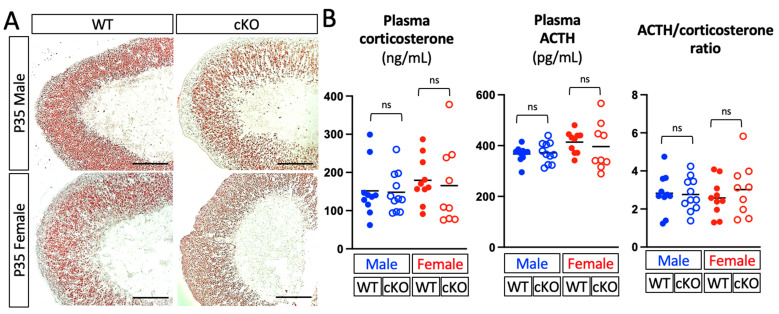
Phenotypic analyses of Dhcr24 cKO mice. (**A**) Oil Red O staining showing lipid droplets in the adrenal glands of euthyroid P35 mice. (**B**) Plasma levels of corticosterone, ACTH, and the ACTH/corticosterone ratio in euthyroid P35 mice. Each dot (WT) and circle (cKO) represents data from one mouse. Data are shown with the mean. Scale bars, 210 µm. WT: wild-type mice, cKO: Dhcr24 conditional knockout mice. ns, no significant difference.

Because DHCR24 is the key enzyme that controls cholesterol synthesis, it is possible that the de novo synthesis of cholesterol is affected in adrenocortical cells in *Dhcr24* cKO mice and then further alters the subsequent steroidogenesis. Phenotypic analyses using Oil Red O staining and a hormone assay showed that the oil accumulation was not affected in adrenal glands at the histological level in cKO mice (Figure 6A); the corticosterone and ACTH levels in blood circulation and the ACTH/corticosterone ratio were also unchanged in both sexes of the cKO mice (Figure 6B). It is important to note that these mice were euthanized by carbon dioxide inhalation, which is considered to be a stressor that leads to elevated corticosterone secretion [20]. The comparable stimulated corticosterone and ACTH levels in cKO mice suggest that the lack of de novo synthesized cholesterol does not significantly affect steroidogenesis under certain stress levels, such as the stress caused by CO_2_ euthanasia. This result is consistent with the expression characteristic of DHCR24 in which it is specifically expressed in the inner cortex or X-zone, the area that is not considered a primary contributor to the steroidogenic function of the adrenal gland cortex. However, the de novo synthesized cholesterol might still be detrimental to chronic stress response, especially some genes involved in cholesterol synthesis (e.g., *Sqle* and *Hsd17b7*) has been reported to be upregulated in the mouse adrenal gland after long-term ACTH administration [21], with the recent identification of an *Abcb1b*-positive subpopulation in the adrenal gland inner cortex [14].

## 3. Discussion

Although the *Dhcr24* cKO mice did not show any strong phenotypes that were examined in this study, our data demonstrated that in the mouse adrenal gland, DHCR24 was specifically expressed in the inner cortex, with temporal and spatial expression patterns that were very similar to those of the X-zone marker gene 20αHSD, which has a sexually dimorphic expression pattern. The major difference between DHCR24 and 20αHSD is that there are DHCR24-positive cells in the adrenal glands of parous female mice, but there is no X-zone in these mice because it regresses during pregnancy [13]. This zonal-restricted expression of DHCR24 indicates that most adrenocortical cells in the mouse adrenal gland do not synthesize cholesterol locally because of the lack of DHCR24. We also found that the deletion of DHCR24 in steroidogenic cells did not lead to major histological and functional changes in the adrenal gland, at least under euthyroid conditions. Although a few subpopulations in the adrenal gland inner cortex have been reported [11,14] along with evidence showing the origin [22,23,24] and the developmental timeline [18] of the X-zone, more studies are needed to discover the physiological function of cells in the mouse adrenal gland inner cortex.

Different sources or supply routes of cholesterol ensure the high demand for cholesterol for steroidogenesis in steroid hormone-producing cells. Other than (1) taking up high- and low-density lipoproteins from the blood circulation and (2) deriving cholesterol from cholesterol esters stored as lipid droplets, synthesizing cholesterol de novo in the endoplasmic reticulum is another way steroidogenic cells obtain cholesterol [2,25]. The restricted spatial expression of DHCR24 in the mouse adrenal inner cortex with the finding of normal corticosterone levels in the *Dhcr24* cKO mice suggest that the de novo synthesis of cholesterol is not the major source of cholesterol used for steroid hormone production in the mouse adrenal gland at least for the stress response caused by CO_2_ euthanization. Because the lack of DHCR24 could lead to the accumulation of its substrate desmosterol [26] and the P450scc enzyme is predicted also to use desmosterol to initiate steroid hormone synthesis [27], the lack of DHCR24 may not affect steroidogenesis if desmosterol is used for steroidogenesis as cholesterol. Whether the de novo cholesterol synthesis is important under severe and chronic stress where continuous high amounts of corticosterone production are needed requires further study. It is possible that the *Dhcr24* cKO will start showing phenotypes under certain conditions (e.g., dexamethasone-induced cortex regression, fasting, long-term ACTH administration, etc.).

In addition to its involvement in the mevalonate pathway that controls cholesterol synthesis, DHCR24 has also been linked to many cellular functions and diseases. DHCR24 is also named ‘selective Alzheimer’s disease indicator-1′ or Seladin-1 because of its connection with AD [7]. Patients with AD suffer from massive neuronal death due to apoptosis in both neurons and glial cells [28]. In the brain, DHCR24 is less abundant in the areas affected by AD [7,29]. Over-expression of *DHCR24* in neurons prevents β-amyloid accumulation and oxidative stress. This neuroprotective function that prevents neuronal loss has been seen both in vitro and in vivo [30,31,32,33,34]. Additionally, it has been demonstrated that DHCR24 has a protective effect against apoptosis by inhibiting caspase-3 activity [35,36]. However, the molecular mechanism for the DHCR24-mediated cell protective effect is not fully understood. Our previous study showed that TRβ1 is specifically expressed in the adrenal gland inner cortex, especially in the X-zone [18]. Because T3 treatment has been shown to increase the size of inner-cortical cells and delay their regression [18,37,38], the T3-mediated delayed regression of the X-zone is possibly a direct effect of the T3-induced high expression of DHCR24 in the inner cortex. We are currently using *Dhcr24* cKO mice to determine if DHCR24 contributes to T3-mediated delayed regression.

Because *Dhcr24* is highly expressed in the inner cortex even under euthyroid conditions, it is also possible that DHCR24 confers cell protective effects under euthyroid conditions [27]. Although no significant difference was noticed in *Dhcr24* cKO mice at P35 in both sexes (Figure 6), we are currently examining some earlier developmental stages to determine if deletion of DHCR24 leads to early regression of the X-zone in euthyroid mice. For example, compared with wild-type mice at the same age who usually retain a thin X-zone, male *Dhcr24* cKO mice may lose all 20αHSD-positive cells at P28 [18]. A time-course analysis will show whether or not *Dhcr24* has a role in controlling the normal regression process of the X-zone under euthyroid conditions.

## 4. Materials and Methods

### 4.1. Animals

C57BL/6J mice were purchased from the Jackson Lab. The *Dhcr24^tm1a(EUCOMM)Wtsi^* (C57BL/6 background) mutant mice (*Dhcr24^lacz^*) were obtained from The European Conditional Mouse Mutagenesis Program (EUCOMM). To generate the conditional knockout mice, the *Dhcr24^lacz^* mice were first crossed with the Flp deleter strain (C57BL/6 background, #7089 from Taconic Biosciences, Germantown, NY, USA) to generate the ‘conditional ready’ strain (*Dhcr24^flox^*). Then, the mice were crossed with C57BL/6J mice to remove the Flp. The *Dhcr24^flox/flox^* mice were then crossed with the SF1-Cre mice (gift from Dr. Keith Parker, has been back-crossed to C57BL/6 for more than 10 generations) to obtain the cKO mice (*Dhcr24^flox/flox^; Sf1-Cre*). All mice were housed in a 12:12 h light-dark cycle (lights on at 6 am) with free access to regular rodent chow and water until sample collection. Mice were euthanized between 2 pm and 4 pm using carbon dioxide, followed by decapitation. Tissues were collected immediately and fixed in ice-cold 4% (*v*/*v*%) paraformaldehyde (PFA) in 1X phosphate-buffered saline (PBS) or frozen by liquid nitrogen. All procedures followed the protocols approved by the Institutional Animal Care and Use Committees at Auburn University.

### 4.2. X-Gal Staining

Tissues were fixed in 2% (*v*/*v*) paraformaldehyde in phosphate-buffered saline (PBS) on ice for 20 min and then rinsed with PBS three times. Samples were immersed in 30% (m/v) sucrose at 4 °C on a shaker until tissues were sunk to the vial’s bottom. Samples were embedded into Tissue-Tek O.C.T. compound (Sakura Finetek, Torrance, CA, USA), and 8 μm sections were collected using positive-charged slides. Cryosections were stained using X-gal staining solution [2 mM MgCl_2_, 5 mM potassium ferricyanide (Sigma-Aldrich, St. Louis, MO, USA), 5 mM potassium ferrocyanide (Sigma-Aldrich), 1 mg/mL X-gal (Teknova, Hollister, CA, USA) in PBS] with incubation at 37 °C overnight.

### 4.3. Immunohistochemistry

Tissues were fixed at 4 °C overnight and processed according to standard immunostaining procedures [11]. In short, paraffin-embedded sections were incubated with primary antibodies (DHCR24, #sc-398938, RRID: AB_2832944, 1:100; DHCR24, #sc-390037, RRID: AB_2923495, 1:100; DHCR24, #ab137845, RRID: AB_2923496, 1:100; Tyrosine Hydroxylase (TH), RRID: AB_628422, 1:500; 20αHSD, RRID: AB_2832956, 1:500; CYP2F2, #sc-374540, RRID: AB_10987684, 1:250) followed by appropriate fluorescein-conjugated secondary antibodies. DHCR24 was detected by a biotinylated secondary antibody followed by a fluorescence tyramide [39]. Fluorescent images were obtained using a Revolve 4 microscope (ECHO). ImageJ (https://imagej.nih.gov/ij/, accessed on 14 December 2022) was used for adjusting the brightness and contrast.

### 4.4. Oil Red O Staining

After overnight fixation, tissues were rinsed by ice-cold PBS and then immersed in 30% (*w*/*v*%) sucrose in PBS at 4 °C on a shaker until tissues were sunk to the vial’s bottom. Samples were embedded into Tissue-Tek O.C.T. compound (Sakura Finetek, Torrance, CA, USA), and stored at −80 °C until cryosectioning. Sections (8 μm) were collected using positive-charged slides, rinsed in PBS, and then incubated in Oil Red O solution [0.18% (*w*/*v*%) Oil Red O powder in 60% (*v*/*v*%) isopropanol/ddH_2_O] for 5 min. After washing with ddH_2_O twice for 5 min each, the slides were covered with an aqueous mounting medium. For each group, at least 3 adrenal glands from 3 mice were analyzed.

### 4.5. Quantitative Real-Time RT-PCR (qPCR)

Total RNA from snape-freezing adrenal glands was isolated using the TRIzol reagent (Thermo Fisher, Waltham, MA, USA) according to the manufacturer’s instructions. The reverse transcription was performed with total RNA and SuperScript IV reverse transcriptase (Thermo Fisher, Waltham, MA, USA) with oligo dT primers. The qPCR analysis was performed as described in a previously published study using PowerUP SYBR Green Master Mix (Thermo Fisher, Waltham, MA, USA) [11]. The relative gene expressions were calculated using relative standard curves with *B2m* or *Actb* (Figure 2E, right panel) as the internal control. Primers used for qPCR are listed below: GCCCTTGGTGTCTATGGGTC (forward) and AGCTCGTAGGCAGTGCAAAT (reverse) for *Dhcr24*; GATAGGCCAGGCCATTCTAAGC (forward) and CATTCCCTGGCTTCAGAGACAC (reverse) for *Akr1c18*; CCATTTGTGGACCCAGGTGA (forward) and GGGTCAGTGCATTTTGGAACA (reverse) for *Pik3c2g*; AAGTGCAACGCTTTGCTGAC (forward) and TGAACTCCTGAGGCGTCTTG for *Cyp2f2*; CCTGGATCCTGACGATGTGAA (forward) and ACAGGTGATGCAGCGATAGT (reverse) for *Thrb1*; CTGCCTCCAGACTTCTTTCG (forward) and TTCTTGAAGGGCAGCTTGTT (reverse) for *Cyp11a1*; TATTGACCTGAAGGGGTGGC (forward) and CAGGTGGTTGGCGAACTCTAT (reverse) for *Star*; CAGTGTTCCCAAGGCCTGAACG (forward) and GGCCATCCGCACATCCTCTTTC (reverse) for *Cyp11b1*; GGAGGCCTTTGATAGCACCA (forward) and TTCAGCAGTGCTTTCTCCGT (reverse) for *Hmgcr*; TGCTACGTAACACAGTTCCACCC (forward) and CATGATGCTTGATCACATGTCTCG (reverse) for *B2m*; ATGGAGGGGAATACAGCCC (forward) and TTCTTTGCAGCTCCTTCGTT (reverse) for *Actb*.

### 4.6. Hormone Assays

Mice were exposed to carbon dioxide (~2 min) until they stopped breathing. The blood was then collected at the decapitation site using EDTA-coated tubes. Plasma was stored at −80 °C until use. Hormones were measured using the corticosterone EIA kit (K014-H1, Arbor Assays, Ann Arbor, MI, USA) and the mouse ACTH ELISA kit (NBP3-14759, Novus Biologicals, Centennial, CO, USA) according to manufacturers’ instructions. Five (corticosterone) and 25 (ACTH) microliters of plasma from each mouse were measured for each data point.

### 4.7. Statistical Analysis

The two-tailed unpaired Student’s *t*-test function in Microsoft Excel was used to calculate *p* values. *p* values less than 0.05 were considered statistically significant.

## Data Availability

Not applicable.
